# Interaction between *FKBP5* variability and recent life events in the anxiety spectrum: Evidence for the differential susceptibility model

**DOI:** 10.1371/journal.pone.0193044

**Published:** 2018-02-21

**Authors:** Beatriz Pérez-Pérez, Paula Cristóbal-Narváez, Tamara Sheinbaum, Thomas R. Kwapil, Sergi Ballespí, Elionora Peña, Marta de Castro-Catala, Maria Dolors Riba, Araceli Rosa, Neus Barrantes-Vidal

**Affiliations:** 1 Departament de Psicologia Clínica i de la Salut, Universitat Autònoma de Barcelona (UAB), Barcelona, Spain; 2 Research, Innovation and Teaching Unit, Parc Sanitari Sant Joan de Déu, Fundació Sant Joan de Déu, Barcelona, Spain; 3 Centre for Biomedical Research Network on Mental Health (CIBERSAM), Instituto de Salud Carlos III, Madrid, Spain; 4 Department of Psychology, University of Illinois at Urbana-Champaign, Champaign, Illinois, United States of America; 5 Secció de Zoologia i Antropologia Biològica, Departament de Biologia Evolutiva, Ecologia i Ciències Ambientals, Universitat de Barcelona (UB), Barcelona, Spain; 6 Institut de Biomedicina de la Universitat de Barcelona (IBUB), Barcelona, Spain; 7 Departament de Psicobiologia i Metodologia de Ciències de la Salut, Universitat Autònoma de Barcelona (UAB), Barcelona, Spain; 8 Sant Pere Claver–Fundació Sanitària, Barcelona, Spain; Istituto Superiore Di Sanita, ITALY

## Abstract

**Background:**

Gene-environment interaction (GxE) research has highlighted the importance of investigating the FK506 binding protein 51 (*FKBP5*) gene as a sensitivity gene. However, previous GxE studies with *FKBP5* have not measured the *full* environmental spectrum or applied statistical tests to discern whether the GxE interaction fits better with the differential-susceptibility or diathesis-stress hypotheses. This study examined whether single nucleotide polymorphisms (SNPs) on *FKBP5* gene moderate the association of positive and negative recent life events (LEs) with depressive symptoms, state-anxiety, neuroticism, and social anxiety traits.

**Methods:**

A total of 86 nonclinical young adults were administered psychological measures and were genotyped for five *FKBP5* SNPs (rs3800373, rs9296158, rs1360780, rs9470080 and rs4713916).

**Results:**

Regression analyses indicated significant GxE interactions for social anxiety and neuroticism. The interactions predicting neuroticism fit different models for different SNPs, although the overall effect indicated by the haplotype was consistent with the differential-susceptibility hypothesis: the risk-haplotype group presented higher neuroticism in the presence of more negative LEs and lower neuroticism in the presence of more positive LEs. The GxE interactions for social anxiety were consistent with the diathesis-stress model. The lack of significance in the for-better side for social anxiety might be related to the fact that it mapped onto low extraversion, which is associated with a lower permeability to positive experiences.

**Discussion:**

Findings underscore the importance of testing the differential-susceptibility model in relation to *FKBP5* to adequately characterize its role in healthy and pathological developmental processes.

## Introduction

Gene-Environment interaction (GxE) research has been primarily guided by the diathesis-stress model [1–3], which establishes that individuals carrying genetic-risk variants are more vulnerable to the effect of environmental adversity and thus more prone to develop psychopathology. Therefore, GxE has predominantly focused on the assessment of the negative side of the environment (adversity) and has almost neglected the positive side (supportive experiences). More recently, Belsky & Pluess [4] have underscored the biasing emphasis of GxE on environmental adversities and proposed an alternative hypothesis, a differential-susceptibility to environmental influences. It poses that, due to evolutionary reasons, individuals with different genetic background should differ in the degree to which they are affected by the whole environmental spectrum (from positive to negative) and not only by the degree in which they are affected by adverse environments, as predicted by the diathesis-stress hypothesis [5]. Therefore, more plastic individuals are expected to be more susceptible to both the negative effects of adverse environments and the beneficial effects of positive environments, while less plastic individuals are expected to be less affected by the environment [6].

Although a fast-growing research has recently pointed to the critical role of positive environments [7], the differential-susceptibility hypothesis has been much less employed to investigate GxE on psychopathology. The problem of GxE being exclusively focused on negative environments is that vulnerability and susceptibility can become indistinguishable, since susceptibility, in some cases, only becomes evident when measuring the full environmental spectrum [4]. Failure to distinguish between diathesis-stress and differential-susceptibility models has critical implications, as it could lead to an inadequate characterization of the etiology of mental disorders and resilience processes. Such biased accounts can result in interventions that are based on an incomplete or even erroneous understanding of human development, which in turn can produce inefficacy, iatrogenic effects, and unnecessary economical costs. Indeed, differential-susceptibility research demonstrates that measuring the full environmental spectrum, genetic variants that were robustly consistent with a diathesis-stress pattern and consequently understood as “risk-variants” (e.g., the short allele of 5HTTLPR), may be better conceptualized as genetic susceptibility factors as they match with differential-susceptibility predictions [8].

The gene encoding FK506 binding protein 51 (*FKBP5*), located on chromosome 6p21.31, is a highly interesting target for GxE as it is considered a shared etiological factor underlying stress-related disorders [9, 10]. The *FKBP5* protein, through the inhibition of glucocorticoid receptors activity, promotes the homeostatic regulation of the hypothalamic-pituitary-adrenal (HPA) axis, the principal biological mechanism of the stress response [10, 11]. Several *FKBP5* single nucleotide polymorphisms (SNPs) have been robustly associated with individual differences in the stress response of healthy adults (e.g., prolonged recovery period of the HPA axis after exposure to a stressor and increased glucocorticoid receptor resistance; [10]). *FKBP5* variability has also been shown to be associated with heightened amygdala reactivity in the context of emotional neglect, increased attentional threat bias and differences in hippocampal shape [10–13], and with anxiety-proneness trait levels [14].

As diathesis-stress research highlighted, the interaction of *FKBP5* variability with childhood, but not adult trauma [15], has been found to confer risk for several psychopathological phenotypes [16], including depression [17, 18], psychosis [19, 20], anxiety [21], suicidal attempts [22, 23], aggression [24] and post-traumatic stress disorder [15, 25]. However, Belsky & Pluess [26] suggested that some of these results may in fact represent differential susceptibility effects. For instance, Xie *et al*. [27] found that individuals homozygous for the risk T allele of the rs9470080 had the highest risk of post-traumatic stress disorder if they were exposed to early trauma, but the lowest if they were not exposed. Likewise, Zimmermann *et al*. [28] found that the cumulative incidence of first-episode major depression was the highest for individuals possessing risk alleles of two *FKBP5* SNPs (rs3000377 and rs47139611) if they had experienced severe trauma, but the lowest if they had not. Similar results were found in the interaction between childhood trauma and risk genotypes of four SNPs (rs3800373, rs9296158, rs1360780, rs9470080) in relation to aggression [24]. Finally, in a sample of post-institutionalized adolescents, Vanzomeren-Dohm *et al*. [18] found that the rs1360780 did not moderate the relation between early adversity and depressive symptoms. However, consistent with a differential-susceptibility pattern, it moderated the association between current peer victimization and depressive symptoms for girls carrying the risk T allele.

These GxE studies highlight the importance of investigating the FKBP5 as a sensitivity gene. Nonetheless, to the best of our knowledge, none of them measured the interplay between FKBP5 variability and the full environmental spectrum or applied statistical tests to discern whether this interaction better fits the differential-susceptibility or diathesis-stress models. In addition, most GxE research has focused on the interaction between childhood trauma and *FKBP5* variants, whereas there is scant knowledge about the interaction of *FKBP5* variants with adult recent life events (LEs). Therefore, the aim of this study was to address these gaps by examining whether *FKBP5* stress-related polymorphisms moderate the association between the full spectrum of adult LEs (from positive to negative) and anxious-depressive trait and state measures in nonclinical young adults. On the basis of the diathesis-stress model, it would be predicted that individuals carrying the risk alleles would show higher scores on the anxiety-depression measures when they experienced more negative LEs (as compared with participants homozygous for the non-risk alleles). In the differential-susceptibility model, the same individuals carrying the risk alleles would also show lower anxiety-depression levels when exposed to more positive LEs (as compared with participants homozygous for the non-risk alleles).

## Methods

### Ethics statement

The study was approved by the Ethics Committee of the Universitat Autònoma de Barcelona (Comissió d'Ètica en l'Experimentació Animal i Humana) and conformed to the Helsinki Declaration. The participants in this interview study were over eighteen years of age and had full capacity to consent to participation in research. All participants provided written informed consent and were paid for their participation.

### Participants

Data were collected as part of an ongoing longitudinal investigation examining psychosis risk and resilience in youth adults from the Universitat Autònoma de Barcelona (n = 547; 16.82% male; mean age = 20.6). A smaller subsample was selected to conduct exhaustive interview and laboratory follow-up measurements. Participants with high schizotypy scores were oversampled to ensure that the selected subsample was representative of the unselected sample but still contained enough variance on schizotypy measures (see [29]). The sample analyzed in this study comprised 86 participants with valid data targeted at the third wave assessment (mean age = 24.8; SD = 2.68; range = 22–34 years; 39.53% male). We examined whether scores of the unselected participants assessed in the first screening sample who were not retained for the third follow-up (group 0; n = 461) differed from those of the present sample (group 1; n = 86). Results of multivariate regression analyses showed that, after controlling for sex and age, the effect of group was nonsignificant for the two schizotypy scores used as screening criteria (i.e., positive schizotypy, p = 0.290; negative schizotypy, p = 0.457), indicating that the present sample is unbiased in terms of schizotypy scores.

### Materials and procedure

Participants completed all psychological measures used in this study through an online procedure and provided buccal mucosa on cotton swabs.

#### Psychological measures

All measures showed an internal consistency between good and excellent (see Table 1). Depressive symptoms were evaluated using the 21-item Beck Depression Inventory Second Edition (BDI-II; [30]), which measures the severity of depressive symptoms (last two weeks) on a Likert-scale from 0 to 3. State-anxiety was measured with the 21-item Beck Anxiety Inventory (BAI; [31]), which assesses the intensity of anxiety symptoms over the last week with ratings ranging from 0 (not at all) to 3 (could barely stand it).

**Table 1:** Descriptive statistics of self-reported measures (n = 86).

Measure	Mean	*SD*	α	Observed range		Theoretical range	
				Min.	Max.	Min.	Max.
Depressive symptoms (BDI-II)	5.58	6.41	0.89	0	33	0	63
State-anxiety (BAI)	5.51	5.81	0.89	0	39	0	63
Neuroticism (NEO-PI-R)	74.14	25.28	0.94	29	127	0	192
Social anxiety & phobia (SPAI-B)	21.07	11.97	0.95	0.36	50.17	0	64
Live events impact (LESms)	0.57	0.98	-	-1.50	2.78	-3	3

*Note*. BDI = Beck Depression Inventory-Second Edition; BAI = Beck Anxiety Inventory; Neuroticism = Neuroticism Subscale from NEO Personality Inventory–Revised; SPAI-B = Social Phobia and Anxiety Inventory-Brief form; LESms = Mean Score of the Life Experiences Survey.

Neuroticism was assessed using the neuroticism subscale from the NEO Personality Inventory-Revised (NEO-PI-R; [32]), which captures the five-factor model of personality. It measures 6 facets (anxiety, angry-hostility, depression, self-consciousness, impulsiveness and vulnerability) via 48 items ranging from 0 (strongly disagree) to 4 (strongly agree). Social anxiety traits were measured with the Spanish version of the Social Phobia and Anxiety Inventory-Brief form (SPAI-B; [33]), a 16-item self-report ranging from 1 (never) to 5 (always).

LEs were measured with the Life Experiences Survey (LES; [34]), a self-report assessing the subjective impact of a variety of LEs over the last year. It is composed of two sections: section 1 contains 47 common LEs plus 3 empty spaces for participants to register events not collected in the questionnaire; section 2, specifically designed for university students, contains 10 items related to academic events. Participants rated the perceived impact of experienced events on a 7-point Likert-scale ranging from -3 (extremely negative) to +3 (extremely positive). The LES provides two measures of subjective impact: the Negative Change Score (the sum of the subjective impact ratings with negative values) and the Positive Change Score (the sum of the subjective impact ratings with positive values). In this study we used the LES mean score (LESms), which is the mean of the subjective impact ratings of the experienced events for each individual, including positive and negative ones. Positive scores indicate that LEs had a positive subjective impact.

#### Genotyping

Genomic DNA was extracted using the Real Extraction DNA kit (Durviz S.L.U., Valencia, Spain). The rs3800373, rs929615, rs1360780, rs9470080 and rs4713916 SNPs on *FKBP5* gene were genotyped using TaqMan 5'-exonuclease allelic discrimination assay (Applied Biosystems) via 5 custom assays. All SNPs were in accordance with Hardy-Weinberg Equilibrium.

Haplotypes were estimated using a Bayesian approach implemented with PHASE software [35]. Linkage disequilibrium, which is the tendency of SNPs to be inherited together, was examined by pair-wise comparisons of r^2^ and D’ using Haploview version 4.2 [36]. We computed individual’s haplotypes considering three tag SNPs (rs3800373, rs929615, rs1360780) reported in previous studies (see Table 2 for comparison groups).

**Table 2:** Description of FKBP5 studied variants and comparison groups employed in multivariate regression analysis.

FKBP5 variants description			Empirical background ^a^		Comparison groups		
SNP	Reference Sequence (rs)	Genotypic or Haplotypic combinations (n)	Non-risk allele	Risk allele	Genotype groups considered (*n*)	*n* ^b^	Theoretical approach
SNP1	rs3800373	A/A (39) A/C (39) C/C (8)	A	C	A/A (39) vs. C carriers (47)	86	N/N vs. Rc
SNP2	rs9296158	G/G (39) G/A (41) A/A (6)	G	A	G/G (39) vs. A carriers (47)	86	
SNP3	rs1360780	C/C (36) C/T (42) T/T (8)	C	T	C/C (36) vs. T carriers (50)	86	
SNP4	rs9470080	C/C (35) C/T (42) T/T (9)	C	T	C/C (35) vs. T carriers (51)	86	
SNP5	rs4713916	G/G (44) G/A (39) A/A (3)	G	A	G/G (44) vs. A carriers (42)	86	
HAPL	rs3800373 rs9296158 rs1360780	AGC/AGC (33) AGC/XXX (8) AGC/CAT (35) CAT/CAT (5) CAT/XXX (2) XXX/XXX (3)	AGC	CAT	AGC/AGC or AGC/XXX (41) vs. CAT carriers (42)	83	N/N or N/X vs. Rc

*Note*. N = Non-risk; Rc = Risk carriers; HAPL = Haplotype; XXX & X = Other haplotype combinations (AAC, AAT, CGC, CGT, CAC or AGT).

^a^ Risk and non-risk alleles according to Zannas and Binder (2014).

^b^ Corresponds to the observations accounted in multivariate regression models for the different FKBP5 moderators.

### Statistical method

In order to avoid misinterpreting possible gene-environment association as gene-environment interactions, the effect of *FKBP5* variants on LESms was examined by T-Test comparisons. We also used T-Tests to analyze whether criteria (symptoms and traits) and predictor (LESms) variables were associated with sex.

Multiple regression analyses were conducted separately to examine the main-effects of the genetic (five *FKBP5* SNPs and haplotype) and environmental (LESms) variables, as well as GxE interactions, on the anxiety-depression measures. Given that discarding the presence of nonlinear phenomena masked by a significant linear interaction is an important step to ensure that data are consistent with differential-susceptibility [37], we estimated additional models for all significant interactions to ensure that data are consistent with differential-susceptibility, including X^2^ (LESms^2^) and X^2^Z (LESms^2^ x *FKBP5* variant). Then, simple slope analyses were conducted to test the statistical significance and magnitude of the environmental effects (LESms) over the criteria at the two levels of the *FKBP5* moderators (risk and nonrisk carriers). These analyses were performed using STATA-12 [38].

In order to compute the Diathesis-Stress and Differential-Susceptibility Indices (DE/DS-Indices) recommended by Roisman *et al*. [37], significant interactions were entered in the Web-based program (http://www.yourpersonality.net/interaction/). These indices included the analysis of Regions of Significance on X (RoS X), the Proportion of Interaction (PoI) and the Proportion Affected (PA). RoS on X indicates the specific values of the predictor (LESms) below and above which the regression lines for the two levels of the moderator (*FKBP5* variant) differ significantly in terms of the criterion. If RoS on X is statistically significant for the right and left sides of the environmental measure then differential-susceptibility is supported. The PoI index expresses the proportion of the total interaction represented on the right side of the crossover point, and indicates the area for which the effect of the predictor on the criterion is “for-better”. PoI values between 0.40 and 0.60 represent an interaction-effect highly consistent with differential-susceptibility, whereas values approaching 0 are highly consistent with diathesis-stress. Given that RoS on X and PoI are affected by the range of observations of the predictor (LESms), they should be computed by convention at ±2 *SD* from the mean of the predictor [37].

PA represents the proportion of individuals differentially affected by the interaction, that is, those falling above the crossover point of the interaction. Roisman *et al*. [37] suggested considering differential-susceptibility when PA is above 0.16 and definitely concluding diathesis-stress when PA is below 0.02. Given that PA needs to be computed on the assumption of a normal distribution of the predictor, we used the Shapiro-Wilk test to show that the LESms did not differ from the normal distribution (p = 0.452).

## Results

T-test comparisons revealed that none of the *FKBP5* variants were associated with the LESms. None of the 4 criteria were associated with sex, whereas LESms was higher in women than in men, indicating that women reported a greater positive impact of LEs (t (84) = 2.31, p = 0.023, d = 0.49, 95% CI [0.07, 0.91]).

Multiple regression main-effects analyses showed that genetic variables did not predict criterion measures (all p > 0.11). In contrast, positive LEs predicted lower levels of depressive symptoms (p < 0.001, 95% CI [-3.84, -1.25]), state anxiety (p = 0.027, 95% CI [-2.66, -0.17]), neuroticism (p = 0.011, 95% CI [-12.41, -1.68]) and social anxiety (p = 0.009, 95% CI [-5.85, -0.76]). Regarding GxE interactions, we found 7 significant results (see Table 3). In all cases, additional models showed that neither X^2^ nor X^2^Z (or their combination) were statistically significant (all p > 0.18), confirming the absence of nonlinear phenomena masked by the significant linear-interactions. No significant GxE interactions emerged for depressive symptoms or state-anxiety, although rs9296158, rs1360780 and the haplotype reached a trend towards significance for state-anxiety. The interaction of rs3800373, rs9296158 and the haplotype with LESms predicted both neuroticism and social anxiety, whereas the interaction of 1360780 with LESms only predicted neuroticism. Simple slopes analyses indicated that in all cases the effect of LESms on neuroticism and social anxiety was only significant for those carrying the risk alleles (non-risk carriers, all p > 0.455).

**Table 3:** Regression estimates, significant simple slopes, and differential susceptibility/diathesis–stress indices by criterion domain.

	Regression estimates^a^						Significant simple slopes^b^			Differential-susceptibility/diathesis-stress indices				
Criterion	*b*_o_	*b*_1_	*b*_2_	*b*_3_	*R*^*2*^_*a*_	*p(b*_*3*_*)* ^*c*^	*b*	95% CI	*p*	RoS X		PoI	PA	Cross
										Lower bound	Upper bound			
*Depressive symptoms*														
SNP1	7.455	-2.019	-0.795	-0.730	0.132	0.590	_	_	_	_	_	_	_	_
SNP2	7.489	-1.980	-0.739	-1.035	0.138	0.438	_	_	_	_	_	_	_	_
SNP3	6.541	-1.689	1.098	-1.791	0.140	0.177	_	_	_	_	_	_	_	_
SNP4	8.088	-2.792	-1.898	0.571	0.138	0.670	_	_	_	_	_	_	_	_
SNP5	7.583	-2.595	-1.124	0.599	0.128	0.964	_	_	_	_	_	_	_	_
HAPL	7.436	-2.046	-0.366	-1.212	0.139	0.387	_	_	_	_	_	_	_	_
*State-anxiety*														
SNP1	5.857	-0.394	1.186	-1.898	0.048	0.142	_	_	_	_	_	_	_	_
SNP2	5.756	-0.449	1.476	-2.148	0.057	0.092	_	_	_	_	_	_	_	_
SNP3	5.298	-0.383	2.126	-2.221	0.065	0.078	_	_	_	_	_	_	_	_
SNP4	5.663	-0.944	1.189	-0.869	0.032	0.500	_	_	_	_	_	_	_	_
SNP5	5.482	-0.815	1.671	-1.306	0.042	0.305	_	_	_	_	_	_	_	_
HAPL	5.825	-0.656	1.603	-2.215	0.069	0.096	_	_	_	_	_	_	_	_
*Neuroticism*														
SNP1	76.944	0.013	4.113	-12.464	0.102	0.024*	-12.45	[-19.72, -5.19]	0.001*	-2.076	1.555^d^	0.62	0.37	0.330
SNP2	75.930	-1.153	6.626	-12.819	0.104	0.019*	-13.97	[-21.81, -6.13]	0.001*	-1.045^d^	1.867^d^	0.52	0.30	0.517
SNP3	73.990	-1.939	8.926	-10.909	0.091	0.044*	-12.85	[-20.53, -5.17]	0.001*	-0.878^d^	11.613	0.37	0.21	0.818
SNP4	77.990	-5.759	0.402	-2.163	0.043	0.697	_	_	_	_	_	_	_	_
SNP5	77.971	-5.463	0.383	-3.729	0.048	0.499	_	_	_	_	_	_	_	_
HAPL	76.514	-2.724	7.405	-12.651	0.123	0.024*	-15.38	[-23.60, -7.15]	<0.001**	-1.025^d^	2.287^d^	0.49	0.28	0.585
*Social anxiety & phobia*														
SNP1	20.849	-0.204	5.106	-6.037	0.107	0.021*	-6.24	[-9.67, -2.81]	0.001*	0.358^d^	9.59	0.18	0.10	1.278
SNP2	20.243	-0.951	6.315	-5.610	0.111	0.029*	-6.56	[-10.26, -2.87]	0.001*	0.190^d^	6.505	0.24	0.13	1.126
SNP3	19.428	-1.459	6.918	-4.179	0.108	0.099	_	_	_	_	_	_	_	_
SNP4	20.197	-0.961	5.092	-4.293	0.085	0.098	_	_	_	_	_	_	_	_
SNP5	20.843	-2.171	4.216	-2.415	0.065	0.352	_	_	_	_	_	_	_	_
HAPL	20.689	-0.630	6.213	-5.896	0.098	0.027*	-6.53	[-10.43, -2.62]	0.001*	0.124^d^	5.365	0.27	0.15	1.054

*Note*. SNP1 = rs3800373; SNP2 = rs9296158; SNP3 = rs1360780; SNP4 = rs9470080; SNP5 = rs4713916; HAPL = Haplotype

*R*^*2*^_*a*_ = adjusted R-squared; RoS X = Regions of Significance with respect to X; PoI = Proportion of Interaction index; PA = Proportion Affected index; Cross = Crossover point at which the regression lines intersect.

^a^ The regression equation Y = b_0_ + b_1_X + b_2_Z + b_3_XZ, where X is life events (LESms) and Z is FKBP5 variant.

^b^ In all cases significant simple slopes were only found in the groups of risk-carriers (Z = 1).

^c^ *p* value of the interaction term.

^d^ RoS on X indices falling within 2 *SD* from the mean of the predictor (LESms).

* *p* < 0.05.

** *p* < 0.001.

**Fig 1:**
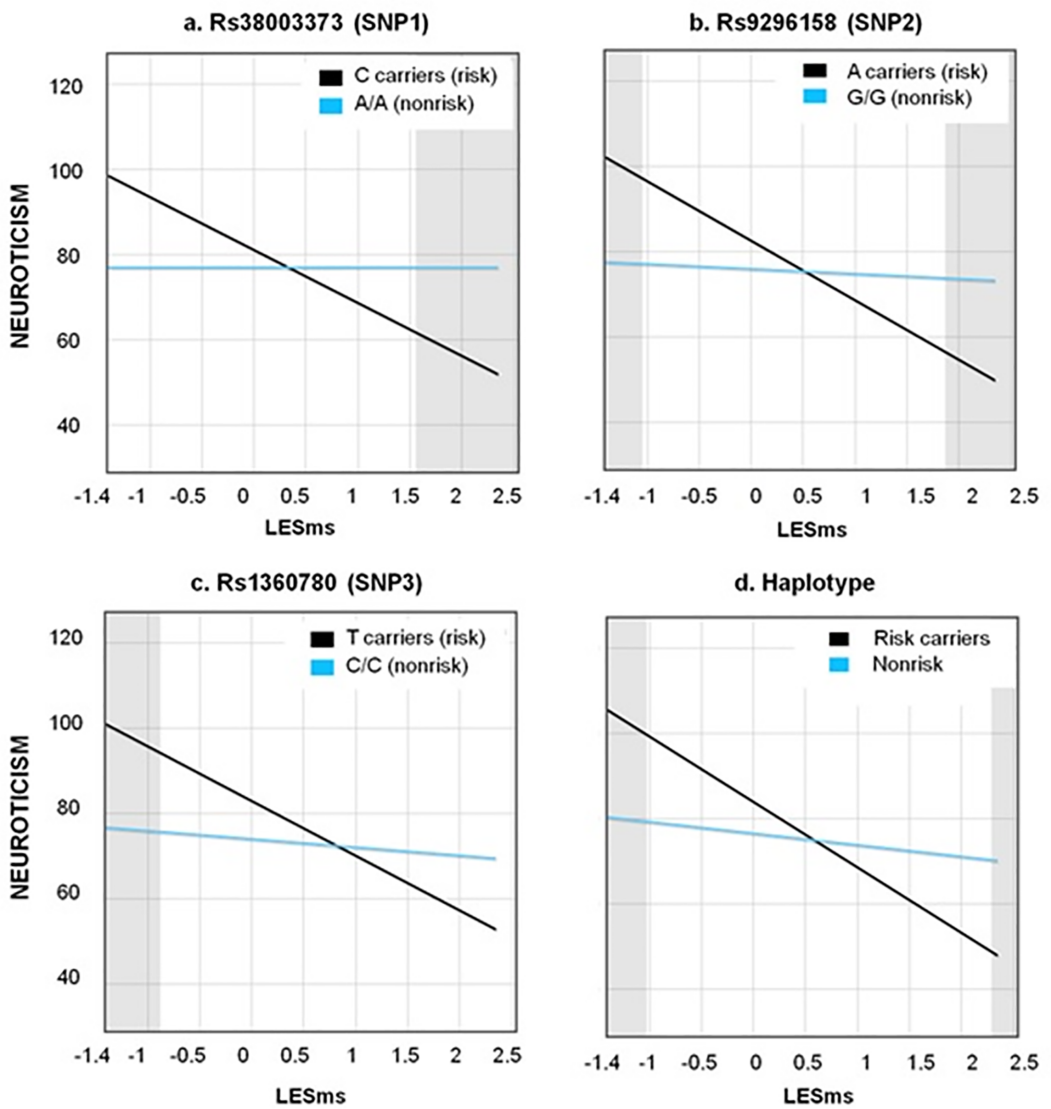
The four significant interactions of FKBP5 variants with recent live events (LESms) for neuroticism, with Regions of significance on X (shaded areas). **Description:** Blue lines represent nonrisk-groups and black lines risk-groups. *Graph (a)* shows a vantage sensitivity effect: the group of individuals carrying the risk-allele (C) only differ from the nonrisk-group (A/A), showing significantly *lower* neuroticism when they experienced more positive LEs. *Graphs (b) and (d)* demonstrate a differential-susceptibility effect: risk-groups, with respect to nonrisk-groups, have significantly *higher* neuroticism if they experienced more negative LEs, as well as significantly *lower* neuroticism if they experienced more positive LEs. *Graph (c)* shows a diathesis-stress effect: the risk-group differed from the nonrisk-group, showing significantly *higher* neuroticism when they experienced more negative LEs. All graphs were plotted at 2 *SD* from LESms (range; -1.401, 2.532).

Table 3 also shows the DE/DS indices. Concerning neuroticism, the RoS on X analysis revealed vantage-sensitivity for rs3800373 in favor of risk-carriers, that is, individuals carrying risk-alleles (C/C or A/C) only differed from those carrying the non-risk alleles (A/A) in that they showed less neuroticism when experiencing more positive LEs. PoI > 0.60 and PA > 0.16 also supported the RoS on X analysis, highlighting the importance of the “for-better side” in this interaction. The interaction of LESms and rs1360780 on neuroticism was consistent with a diathesis-stress pattern evaluated with the RoS on X and the PoI indices (PoI 0.40 > 0.60), that is, the risk group showed higher neuroticism when experiencing more negative LEs. However, it was consistent with differential-susceptibility as evaluated with the PA, which indicated that 21% (criterion > 16%) of individuals were differentially affected by the interaction. Regarding rs9296158 and the haplotype, all DE/DS-indices converged to be highly consistent with a differential-susceptibility pattern. Individuals carrying either risk alleles or the risk-haplotype had higher neuroticism when experiencing more negative LEs as well as lower neuroticism when experiencing more positive LEs as compared with non-risk carriers (see Fig 1).

**Fig 2:**
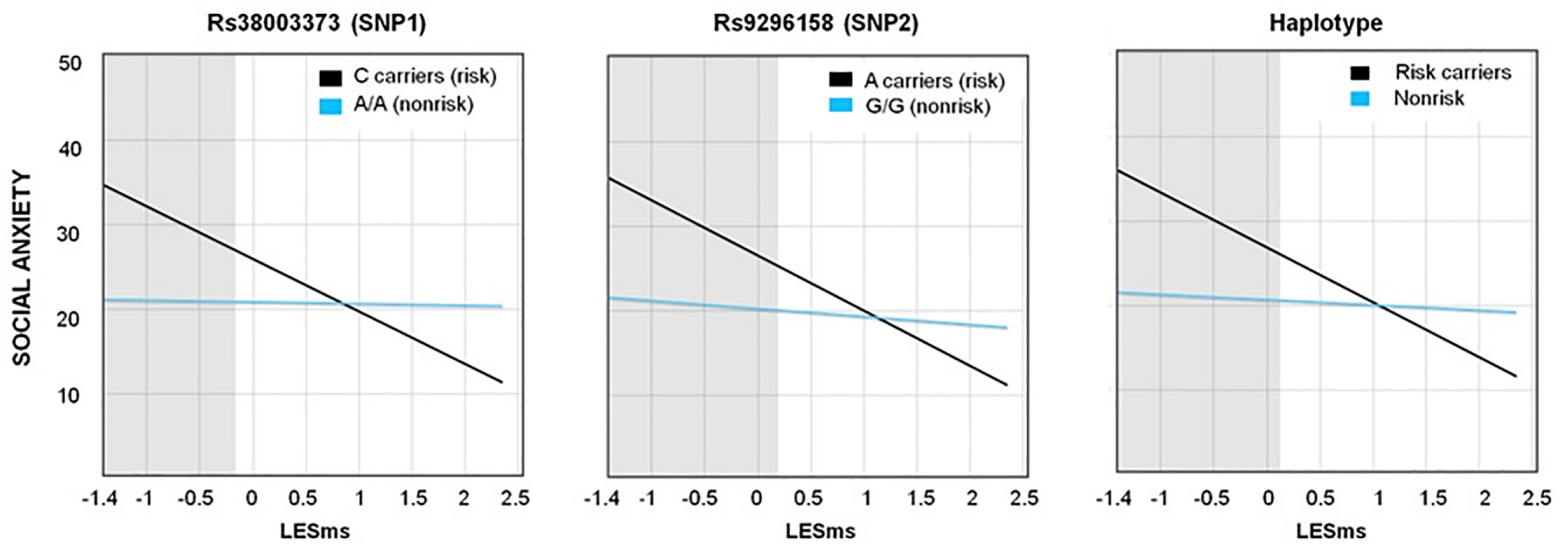
The three significant interactions of FKBP5 variants with recent live events (LESms) for social anxiety, with Regions of significance on X (shaded areas). **Description:** Blue lines represent nonrisk-groups and black lines risk-groups. All 3 graphs demonstrate a diathesis-stress effect: the risk-groups only differed from the nonrisk-groups by showing significantly *higher* social anxiety if they experienced more negative life events. All graphs were plotted at 2 *SD* from the mean of LESms (range; -1.401, 2.532).

Regarding social anxiety, rs3800373, rs9296158 and the haplotype also showed significant interactions with LESms but, unlike neuroticism, all DE/DS-indices converged to be highly consistent with a diathesis-stress pattern; that is, individuals carrying the *FKBP5* risk alleles only differed from those carrying the non-risk alleles in showing higher social anxiety when they experienced more negative LEs (see Fig 2).

### Additional analyses

We performed exploratory analyses partialing out BDI-II and BAI variables to estimate the impact of depressive-anxiety symptoms on the significant interactions. Specifically, we partialed BDI-II and BAI variables out of the analysis of the LES x *FKBP5* interaction. The results showed that the interaction with rs1360780 (diathesis-stress) lost statistical significance (p = 0.26), whereas all the other interactions involving a for-better side (rs3800373, 9296158 and haplotype) remained almost the same (only one dropped statistical significance to p = 0.06; see S1 Table). This may suggest that depressive-anxiety symptoms may be deeply involved in the for-worse effect found for rs1360780, but may not be involved in the for-better effects for rs3800373, rs9296158, and the haplotype. This would be consistent with research showing that the changes induced by negative LEs on neuroticism occur via an increase in anxiety-depressive symptoms, which is not the case for positive LEs [39]. Also, this would fit with evidence indicating that rs1360780 is one of the most relevant SNPs involved in the stress response [10]. However, this should be interpreted cautiously in the current study because of the post hoc nature of the analyses and because the BDI-II and BAI variables were entered in the model only as main effects. Following the suggestion of a reviewer, we reran all of the analyses in S1 Table using the recommendations by Keller [40], who suggested that in order to properly control for potential confounders, all the covariate-by-environment and the covariate-by-gene interaction terms should be entered in the same model that tests the gene-by-environment interaction term (see S1 Table). In this case, none of GxE interactions remain significant.

## Discussion

The present study examined whether *FKBP5* variability moderated the association of LEs with depressive symptoms, state-anxiety, neuroticism, and social anxiety. To the best of our knowledge, this is the first GxE study examining *FKBP5* variability in the full spectrum of environmental exposures and directly testing differential-susceptibility versus diathesis-stress models. Significant GxE interactions emerged for neuroticism and social anxiety, whereas no interactions were found regarding depressive symptoms and state-anxiety.

Different moderation profiles were found for neuroticism: rs3800373 was consistent with a vantage-sensitivity effect, rs9296158 with differential-sensitivity, rs1360780 with diathesis-stress and, importantly, the haplotype (that accounted for all the accumulative genetic variance of those SNPs) was consistent with differential-susceptibility. The overall differential-susceptibility pattern was consistent with the cross-sectional study by Pluess *et al*. [41], who found the same pattern for homozygous subjects on the stress-related risk allele of the 5-HTTLPR gene and suggested that neuroticism could be a stable or unstable trait depending on the genotype. Furthermore, a recent non-genetic longitudinal study evidenced long-lasting changes on neuroticism induced by LEs, such that positive LEs *decreased* neuroticism scores whereas negative LEs *increased* them. Interestingly, the relation was mediated by depressive and anxiety symptoms for negative but not for positive LEs, which had the strongest effect [39].

Unlike the differential-susceptibility effect in neuroticism, all significant interactions for social anxiety were consistent with a diathesis-stress pattern. This discrepancy might be explained by the interplay of social anxiety with neuroticism, extraversion, and resilience. Social anxiety is a combination of high neuroticism and low extraversion; actually, the genetic factors that influence those dimensions account entirely for the genetic liability to social anxiety [42]. Interestingly, both personality dimensions, which are moderately heritable and also shaped by LEs, are linked to resilience [39, 43, 44]. High extraversion is associated with high positive affect, social support, and enhanced learning from positive reinforcement [32, 45]. The impact of positive LEs on building-up resilience, which is stronger than the impact of negative LEs, is mediated by decreases in neuroticism and increases in extraversion [44]. Therefore, given that social anxiety is defined by low extraversion and this involves a lower permeability to positive experiences, it is not surprising that, unlike neuroticism, the for-better side of the interaction indicating benefit from positive experiences was not significant. This resonate with the notion that additional environmental/genetic factors influencing extraversion would be involved in social anxiety. In this sense, the dopaminergic *DRD4* gene might be an interesting candidate as it is linked to extraversion and has been proposed as a sensitivity marker [46, 47].

In the diathesis-stress model, being affected by environmental adversities is equivalent to being vulnerable, whereas being unaffected is equivalent to being resilient. By contrast, in the differential-susceptibility model, putatively vulnerable individuals are considered to be plastic in a for-better-and-for-worse pattern, whereas the putatively resilient individuals are thought to be “unmalleable” to both supportive and adverse environments [26]. It seems possible thus to distinguish two different GxE processes underlying a resilient outcome (i.e., overcoming adversity) depending on the degree of environmental sensitivity. The first would be a relatively stable resilience process derived from being unaffected by environmental influences (either positive or negative) and would characterize individuals who are considered “resilient” under both models. The second would be a highly dynamic process arising from enhanced responsiveness to environmental influences and would characterize individuals considered to be “vulnerable” (diathesis-stress) or “plastic” (differential-susceptibility). In this dynamic process, the detrimental effects of adversity would be compensated by benefiting from the positive effects of subsequent supportive experiences. The plausibility of these processes appears to be supported by research suggesting that individuals carrying “sensitivity” genes benefit the most from certain therapeutic interventions [48].

Previous studies reporting GxE interactions on adult depressive outcomes mostly evaluated the effect of distant and severe childhood adversities [10]. In those studies, the most replicated GxE interaction was found for rs1360780, which has also been shown to mediate childhood trauma interactions via epigenetic processes [49, 50]. Unlike previous work, the present study evaluated the effect of common (not necessarily traumatic) and recent LEs in early adulthood. Therefore, it is attractive to speculate that the lack of significant *FKPB5* x adult LEs interactions on depressive symptoms found in this study is partially related to the fact that epigenetic changes that mediate the association of early-life adversity with adult depression are no longer (or less dramatically) induced by the impact of negative (though not necessarily severe) LEs in adulthood. Regarding state anxiety, the measure used in our study is largely characterized by somatic complaints and might not be as sensitive to *FKPB5* x LEs interactions as the trait and social component measures.

The strengths of this study include the estimation of a haplotype that increases the power to detect genetic associations [51] and the use of specific statistical tests to formally investigate the pattern of GxE interactions [37]. Limitations of the study include the cross-sectional design, and therefore the speculative nature of any causal inference; the use of self-report measures, which entail memory biases; and the relatively small sample size, which limited the statistical power to detect significant results. It is important to note that we did not control for multiple testing in the present study given that our analyses were limited to testing a priori hypothesized relationships and the serious limitations related to post hoc alpha adjustment [52]. However, following a Reviewer’s suggestion about controlling for covariate interaction terms as recommended by Keller [40], we have included a table containing these results as supplementary material. Finally, we also estimated additional models for all significant interactions to ensure that data were consistent with differential-susceptibility. In this sense, although we used the three commonly used tests of differential-susceptibility proposed by Roisman et al. [37] that have been extensively used in the GxE field, recent methodological work [53] suggests that these tests present relevant limitations that can be minimized with novel approaches. More empirical research is needed to analyze the performance and limitations of Roisman’s guidelines.

From a clinical standpoint, this avenue of research with the *FKBP5* gene may have interesting implications. For instance, there have been efforts to develop drugs that block *FKBP5* activity in order to modify the risk for the development of stress-related disorders [10, 16] based on research conducted within a diathesis stress framework. Further examination of whether FKBP5 fits better a differential susceptibility model might be relevant to inform whether interventions aimed at blocking *FKBP5* activity in individuals carrying ‘risk’ genotypes might have the undesirable effect of altering a natural resilience mechanism: the potential of benefitting from positive and supportive environments.

To conclude, our results indicated that the interactions predicting neuroticism fit different models for different polymorphisms, although the overall effect indicated by the haplotype was consistent with the differential-susceptibility hypothesis. The interactions predicting social anxiety (rs3800373, rs9296158 and haplotype) were consistent with the diathesis-stress model, possibly reflecting that the low extraversion component of social anxiety is associated with a lower permeability to positive experiences.

## Supporting information

S1 Table Comparison of the interaction terms and explained variance between the original and the post-hoc regressive models for the neuroticism and the social anxiety statistical significant interactions. (DOCX)
