# Supplementary material for: Interaction between FKBP5 variability and recent life events in the anxiety spectrum: Evidence for the differential susceptibility model
Source: PLoS One. 2018 Feb 21;13(2):e0193044. doi: 10.1371/journal.pone.0193044 (PMC5821376; doi:10.1371/journal.pone.0193044)
Supplement: S1 Table — (DOCX) [file pone.0193044.s001.docx]

**S1 Table. Comparison of the interaction terms and explained variance between the original and the post-hoc regressive models for the neuroticism and the social anxiety statistical significant interactions.**

| Criterion |  | Original model^a^ | | | Post-hoc model^b^ | | | Post-hoc model^c^ | | |
| --- | --- | --- | --- | --- | --- | --- | --- | --- | --- | --- |
|  |  | *b*(XZ) | *p*(XZ) | R^2^_a_ | *b*(XZ) | *p*(XZ) | R^2^_a_ | *b*(XZ) | *p*(XZ) | R^2^_a_ |
| **Neuroticism** | Ref. Sequence (rs) |  |  |  |  |  |  |  |  |  |
| SNP1 | rs3800373 | -12.464 | 0.024* | 0.102 | -8.883 | 0.030* | 0.52 | -6.697 | 0.131 | 0.53 |
| SNP2 | rs9296158 | -12.819 | 0.019* | 0.104 | -8.448 | 0.040* | 0.51 | -5.831 | 0.184 | 0.53 |
| SNP3 | rs1360780 | -10.909 | 0.044* | 0.091 | -4.645 | 0.256 | 0.50 | -4.783 | 0.274 | 0.51 |
| Haplotype | rs3800373 |  |  |  |  |  |  |  |  |  |
|  | rs9296158 | -12.651 | 0.024* | 0.123 | -7.900 | 0.062 | 0.51 | -6.247 | 0.185 | 0.52 |
|  | rs1360780 |  |  |  |  |  |  |  |  |  |
| **Social anxiety** |  |  |  |  |  |  |  |  |  |  |
| SNP1 | rs3800373 | -6.037 | 0.021* | 0.107 | -4.750 | 0.046* | 0.27 | -1.806 | 0.473 | 0.31 |
| SNP2 | rs9296158 | -5.610 | 0.029* | 0.111 | -4.047 | 0.085 | 0.27 | -1.472 | 0.554 | 0.32 |
| Haplotype | rs3800373 |  |  |  |  |  |  |  |  |  |
|  | rs9296158 | -5.896 | 0.027* | 0.098 | -4.135 | 0.089 | 0.26 | -1.342 | 0.609 | 0.32 |
|  | rs1360780 |  |  |  |  |  |  |  |  |  |

*Note. b*(XZ) = coefficient of the interaction term; *p(XZ) = p* value of the interaction term; *R^2^_a_ =* adjusted R-squared.

^a^ The original regressive model for testing interaction effects.

^b^ The original model after including the Beck Depression Inventory-II (BDI-II) and the Beck Anxiety Inventory (BAI) as covariates in the model.

^c^ In order to examine the effect of the LESms x *FKBP5* interaction following Keller [41], all simple effects (LESms, *FKBP5* variability, BDI-II, BAI) and interaction effects between the covariate and the genetic and environmental variables (BDI-II x LESms and BDI-II x *FKBP5* variability; BAI x LESms and BAIms x FKBP5 variability) were entered in the same model.

* *p* < 0.05
